# On the evolutionary origins of differences in sexual preferences

**DOI:** 10.3389/fpsyg.2015.00628

**Published:** 2015-05-21

**Authors:** Daniil Ryabko, Zhanna Reznikova

**Affiliations:** ^1^INRIA Lille, Villeneuve d'AscqFrance; ^2^Laboratory of Behavioural Ecology of Animal Communities, Institute of Systematics and Ecology of AnimalsNovosibirsk, Russia; ^3^Novosibirsk State UniversityNovosibirsk, Russia

**Keywords:** fisher runaway, sexual preferences, evolution of behavior, mate choice, estimation of attractiveness

## Abstract

A novel explanation of the evolutionary process leading to the appearance of differences in sexual preferences is proposed. The explanation is fully general: it is not specific to any particular type of sexual preferences, nor to any species or population. It shows how different sexual preferences can appear in any large group-living population in which sexual selection is sufficiently strong in each sex. The main idea is that the lack of interest toward a member of the opposite sex may be interpreted as a signal of popularity, and thus of reproductive success. It is then boosted by the Fisher runaway process far beyond the point where it becomes costly, resulting in a generalized trait—lack of interest toward the opposite sex. If the interest diverts toward other targets then different sexual preferences emerge. This hypothesis is placed into the context of other works on different sexual preferences in animals; supporting evidence from the literature is reviewed and additional research needed to confirm or refute the hypothesis in any given species is outlined.

Чем меньше женщину мы любим,

Тем легче нравимся мы ей

И тем ее вернее губим

Средь обольстительных сетей. ^1^

A.S. Pushkin, *Eugene Onegin*, 1825–1832

## 1. Introduction

Sexual preferences differ. This simple fact, documented across a bright variety of species and to which we are now accustomed, remains puzzling for evolutionary biologists. Indeed, an individual that redirects its reproductive capacity away from conspecifics of the opposite sex capable of reproduction seems to directly destroy its fitness. This phenomenon has attracted a great deal of attention in recent decades—perhaps, not the least because it is manifest in our own species—resulting in many hypotheses that attempt to explain it (Bagemihl, 1999; Bailey and Zuk, 2009; Poiani, 2010; Bierbach et al., 2013; Scharf and Martin, 2013; Rice et al., 2013). However, most authors agree that the paradox still remains unsolved (see Poiani, 2010, for a review). In this paper we propose a simple general hypothesis that explains evolutionary mechanisms leading to the appearance of sexual preferences other than toward conspecifics of the opposite sex capable of reproduction. By “general” we mean that it is not tied to the physiological or other specifics of any given species, nor is it limited to some particular kind of sexual preference. Instead, a single qualitative condition is required for the emergence of different sexual preferences. This condition is that *the sexual selection is mutual*, that is, both females select males and males select females.

We begin with a brief note on terminology. By *preference* we mean the likelihood of choice: for example, an individual prefers partners of the same sex means that, shall a choice be available, this individual is more likely to choose a partner of the same sex rather than of the opposite one. Thus, we make no attempt to study or interpret animals' perception of the choice or, more generally, their mental states. The subject of interest is *different sexual preferences (DSPs)*, which means sexual preferences for anything different from the conspecifics of the opposite sex capable of reproduction. Examples include preferences for conspecifics of the same sex, for members of different species, for nothing in particular (asexuality), for conspecifics of the opposite sex that are either too young or too old to reproduce, for inanimate objects, etc. Furthermore, *attractiveness* of an individual is its likelihood of being chosen. In this work we only speak about attractiveness in the context of reproductive opportunities; thus, in this paper, attractiveness of an individual is its likelihood of being chosen on average by the members of the opposite sex capable of reproduction present in the population. Finally, *popularity* refers to the realized attractiveness, that is, to the frequency of being chosen.

The main idea of the proposed hypothesis is as follows. In a sex subjected to sexual selection some individuals become more attractive than others. If the selection is acting upon the other sex as well, the more an individual is attractive, the choosier it can allow itself to be. Being choosy, it becomes “hard to get:” it demonstrates a lack of interest toward many or even most of the members of the opposite sex in the population. Thus, any member of the population can experience the lack of interest of someone of the opposite sex toward itself, and interpret it as choosiness and thus as popularity of the disinterested individual. The next logical step is the Fisher runaway process triggered by this sign of popularity. The Fisher runaway (see Section 2.1) is the explosive development of any given trait on which sexual selection starts to act. This development often proceeds well-beyond the point where the trait acquires a reproductive cost, and only stops when this cost balances the benefits provided by the positive selection on the trait. Here the trait is the selective lack of interest (choosiness). Its emergence as a sign of popularity triggers sexual selection of the most disinterested one (the choosiest). By means of the runaway, what starts as only a selective lack of interest, develops into the generalized trait of the lack of interest in members of the opposite sex capable of reproduction, and thus into DSPs. The cost incurred is that of reproductive opportunities missed due to the lack of interest, and the benefit is that provided by the popularity of the trait.

It is important to observe that the individuals that develop DSPs by the process described do not decrease their fitness: under stable conditions, their reproductive output does not differ from the average. Thus, complete aversion to reproductive opportunities cannot develop in this way. However, further radicalization of DSPs is possible if external forces help the Fisher runaway proceed further, as we explain below.

The rest of the paper is organized as follows. The remainder of this section provides a brief overview of the literature on the evolutionary origins of DSPs. Section 2 is devoted to the problem of estimating the attractiveness of potential mates, which is crucial for the proposed hypothesis; it also describes the phenomenon known as Fisher runaway process. Section 3 presents the main result: the hypothesis we advance to explain the evolutionary origins of DSPs. Section 4 presents supporting evidence and describes how the validity of the proposed hypothesis for any given population can be proven or refuted.

### 1.1. Existing explanations of different sexual preferences

Before giving a brief overview of the existing explanations of DSPs, we note that a related phenomenon widely studied in the literature is what can be called *differently targeted sexual behaviors (DTSPs)*. This means sexual behaviors targeted at anything different from the conspecifics of the opposite sex of reproductive age. DTSPs can be established by direct observation, whereas establishing that a DSP is present in a population may require special experiments or longer observations. For example, same-sex sexual behaviors have been observed in hundreds of species of different taxa, including insects and arachnids (Scharf and Martin, 2013), in many of which same-sex sexual preferences have not been established. In particular, in some species males have been observed copulating with inanimate objects loosely resembling female conspecifics, in which case one can hardly speak about a DSP. Moreover, it seems inadequate to try to find a single explanation for such behaviors and for life-long, genetically determined DSPs.

There is no shortage of examples of species in which DSPs have been documented. This includes not only same-sex sexual preferences in various mammals and birds (Bagemihl, 1999; Poiani, 2010), but also, for example, asexuality in rats (Portillo et al., 2006), and rams (Roselli et al., 2002), not to mention the multitude of different preferences in humans (Bullough and Bullough, 2014).

A large body of literature exists on DTSBs and DSPs, mainly focussing on same-sex behaviors. While most authors agree that the questions of why and how such behaviors have appeared in the course of evolution is still enigmatic, several hypotheses of various generality have been advanced. Without attempting to give a comprehensive overview (which is not necessary given those available, such as Bagemihl, 1999; Kirkpatrick, 2000; Roughgarden, 2004; Bailey and Zuk, 2009; Poiani, 2010), and point out some of the limitations of this hypotheses.

It should also be noted that most of the literature on the subject is devoted to same-sex behaviors or preferences, ignoring other preference targets, even though some of the explanations advanced are applicable more generally. We find that such a restriction is not necessary for the explanation we propose, and thus consider the phenomenon of DSPs in all its generality.

#### 1.1.1. Genetic mistake

Perhaps the simplest hypothesis for explaining DSPs is that of a recurrent mutation, that is, of a “genetic mistake.” Thus, a different preference would be maintained in a population due to a recurrent mutation alone (Poiani, 2010). While the argument seems conceptually simple, and some traits may indeed persist in a population at a low frequency due to recurrent mutations, it seems unsuitable to explain a phenomenon that is present in many distant species. Indeed, the possibility that an essentially random mechanism with an unlimited outcome space and high entropy should produce similar results in many almost independent trials is statistically negligible.

#### 1.1.2. Sexual conflict

Sexual conflict is a conflict between the evolutionary interests of individuals of the two sexes. More specifically, sexual conflict arises in a situation when a genetic trait is detrimental to one sex but is advantageous to the other. Early notions of sexual conflict are implicit in the work of Darwin (1871). The concept came into focus in the 1970s (Downhower and Armitage, 1971; Dawkins and Krebs, 1979), with a large body of subsequent work concentrating mainly on the evolution of female mate choice (Holland and Rice, 1998; Cameron et al., 2003; Cordero and Eberhard, 2003; Clutton-Brock and McAuliffe, 2009).

As a hypothesis for explaining DSPs, sexual conflict means that the genes that in one sex result in a DSP, to the other sex confer some reproductive benefit. Thus, in humans male homosexuality could be associated with female fecundity (Ciani et al., 2008), that is, the same genes that result in homosexuality in the male would increase fecundity in the female. The latter hypothesis has found some support in a recent study on a Samoa population (VanderLaan et al., 2012).

Note that, while this hypothesis may indeed explain the appearance of a certain different preference in a single species or population, the same critique applies as to the “genetic mistake” hypothesis: if a certain gene is detrimental (say) to the male but is advantageous to the female, then an adaptation should develop over time that switches it off in the male. It is of course possible that a certain species is at present at the point when such an adaptation is yet to develop. However, that several distant species are at that same point simultaneously is highly unlikely.

#### 1.1.3. Kin selection

Kin selection theory (Hamilton, 1964a,b) explains a decrease in individual fitness by the increase of fitness an individual provides to its relatives. Applied to the phenomenon in question, this opens the possibility that individuals with DSPs may convey some reproductive benefit to close relatives in the form of alloparental care. This means that they sacrifice their chances to reproduce in order to provide some direct benefits to the relatives (Emlen, 1982; Solomon and French, 1997; Clutton-Brock, 2002). The kin-selection hypothesis has been among the early explanations suggested for the evolution of homosexuality in humans. Thus, it was suggested that homosexuals would altruistically forgo reproduction to assist the offspring of relatives (Wilson, 1975; Weinrich, 1982); or, more specifically, children could be manipulated by parents to forgo reproduction, become homosexual, and assist the offspring of relatives (Trivers, 1972, 1974). However, despite the fact that this is one of the oldest hypotheses proposed for the evolution of DSPs, so far it has found little more than anecdotal support (Kirkpatrick, 2000), and in fact some studies show empirical evidence to the contrary (Bobrow and Bailey, 2001). One notable exception is a Samoan study by Vasey et al. (2007), which finds some empirical support for this hypothesis for the population in question, but notes that further research is needed to conclusively establish its validity.

Another explanation using the kin selection argument stipulates that the benefit provided to the relatives by those individuals that do not reproduce (or reproduce less) may be simply the increased availability of the resource, rather than alloparental care (Poiani, 2010). For example, dominant individuals may prevent subordinates from reproducing, and exploit the increased reproductive opportunities themselves. Members of a population may “queue” for reproduction, waiting for the dominant to die or leave, and taking his place (for reviews see: Bergmüller et al., 2007; Gardner and Foster, 2008). However, in this case it is disadvantageous for a parent to produce an offspring that would sacrifice its chances to reproduce. In other words, there is no point producing an offspring that would neither provide any help to the parent (e.g., to raise other offspring) nor reproduce itself. This indicates that there should be no strong genetic predisposition for adopting such subordinate roles, making this explanation unsuitable for explaining those DSPs that are genetically determined.

#### 1.1.4. Mating as a signal of attractiveness

Although this explanation applies only to DSTBs and not DSPs, it bears some important similarities to the explanation we propose here. It purports that mating behavior itself can be interpreted as a sign of popularity, and thus it may be beneficial to express it by other means than mating with conspecifics of the opposite sex, leading to such DSTBs as mating with members of different species or same-sex conspecifics (Schlupp et al., 1994; Bierbach et al., 2013). Thus, similar to the explanation we propose, here in the origin of the DSTBs lies the problem of estimating the attractiveness, and, specifically, a particular sign of popularity. This hypothesis is further explained below in Section 2.1.

## 2. Attractiveness and its estimation

In the presence of sexual selection, it is advantageous for an individual to maximize the attractiveness of its offspring. Let us consider what “attractiveness” means here, using female mate choice as an example (the same argument applies the other way around). The goal is to maximize the chances of one's offspring to reproduce in the next generation. To achieve this, a female is looking for a partner whose chance to reproduce (in this generation) is as high as possible. This means that, as compared to other males present in the population, he is more likely to be chosen by other females: he is attractive. Thus, to find whether a given male is attractive, ideally the female of our example would query how likely he is to be chosen by each other female present in the population; she would also do the same evaluation for all the rest of the males. Based on these, she would be able to judge whether he is indeed attractive. Finally, if sexual selection acts in the opposite way as well (males select females), she would also need to evaluate her own attractiveness and that of the rest of the females. With all this information she would be able to determine the appropriate mating choice. While this task might be relatively straightforward in small populations, it is clear that queries of this kind are typically impossible to make in larger populations. Thus, animals have to resort to estimates of attractiveness. Moreover, the role of estimated attractiveness in animals' lives stretches far beyond the mere choice of a mating partner. In particular, parental investment can be influenced by one's own estimated attractiveness (Sanz, 2001) and by that of the chosen mating partner (Kingma et al., 2009). Possible methods for obtaining such estimates are considered in this section.

### 2.1. Attractive features and the fisher runaway process

The so-called Fisher runaway is a process originally proposed by Fisher (1930) as an explanation of seemingly paradoxical evolution of exaggerated traits that do not appear to increase fitness in any obvious way, but only to decrease it. The classical example of such a trait is the peacock's tail, which is costly to maintain and decreases the bird's agility. The explanation is through a positive feedback loop driven by sexual selection. If (say) females select on the most expressed trait in the male, it becomes advantageous for males to possess it. Then it also becomes advantageous for females to select on it, since their offspring, if male, will acquire a reproductive benefit. Their daughters are likely to inherit the selection. Thus, the trait and the selection on it confer a reproductive benefit. The trait itself may be costly. However, it develops up to the point when this cost is counterbalanced by the benefit accrued due to the positive selection on it. Theoretical models of varying generality demonstrating the viability of features that decrease the fitness but are subjected to a positive sexual selection have been developed, and their pertinence have been analyzed both theoretically and empirically (Lande, 1980, 1981; Pomiankowski and Iwasa, 1998; Mead and Arnold, 2004; Kokko et al., 2006).

Thus, Fisher runaway explains the appearance of what can be called *attractive features*. Attractive features can be seen as an *agreement* (obviously, in a figurative sense): if a population *agrees* that a certain feature is attractive, then this feature *is* attractive. Moreover, as explained by the Fisher runaway process, such agreements are easy to establish and hard to break. Easy to establish, because once a small part of the population agrees on the attractiveness of a feature, it already gives a slight benefit for the rest to follow suit. This means that attractive features can appear *at random*, from a random mutation that happens to spread over a small part of the population. (Note, however, that attractive features do not have to appear entirely at random either; they may stem from an honest signal of genetic quality. Nevertheless, once the runaway process starts, the benefits provided by the trait being selected are mostly due to the selection itself, regardless of whether there was an honest signal in the beginning or just a random aberration of the selection process.) Hard to break, because selecting against the agreement (that is, selecting a mating partner that does not possess the feature) disadvantages one's future offspring if the rest of the population follows the agreement and thus does not consider them attractive. This phenomenon is used to explain a variety of often bizarre traits, both physical and behavioral, in a multitude of species(Grafen and Johnstone, 1993; Endler, 1995) including humans (Symons, 1980; Grammer et al., 2003; Gangestad and Scheyd, 2005).

It should be noted that the Fisher runaway process may not spread over a whole population, but only over a part of it. This happens when the population is divided into distinct fractions of bearers of *alternative reproductive tactics* (ART). ART refers to consistent variation in the reproductive behavior (involving, e.g., mating, nesting, fighting) of males or females within one population (Oliveira et al., 2008). In some species populations are divided into fractions consisting of bearers of different ARTs at all times, and these fractions exhibit distinct morphological traits (Gross, 1996; Neff and Svensson, 2013). For example, alternative male reproductive tactics include large fighter males, intermediate-sized males that mimic females, and small sneaker males, were described in a small marine isopod *Paracerceis sculpta* (Shuster and Wade, 1991) and in side-blotched lizards *Uta stansburiana* (Sinervo and Lively, 1996). When more than one ART is present in a population, a trait that is a sign of attractiveness for a bearer of one ART does not necessarily signal attractiveness in bearers of other ARTs. For example, a male may be attractive for extra-pair copulations but not attractive for a pair bond, and vice versa. Thus, rather than spreading over a whole population, a feature of attractiveness may spread only among its fraction that consists of bearers of a certain ART. This process may lead to a further differentiation of the fractions and thus to polymorphism.

### 2.2. Mate-choice copying

A rather direct way to determine the attractiveness of a potential mate is to gather information by observing the choices made by other members of the population. Thus, mate choice can be influenced by another's successful choice. Specifically, a female can consider a male attractive if she observed him being chosen by other females. The number of such observations can be very small (e.g., one or two). The logic of such a choice (though, of course, not necessarily the thought process) is as follows: “If he is chosen by someone right now, this means he is likely to be chosen on average over the population, so I choose him.” This process is known as mate-choice copying (Dugatkin, 1998; Pomiankowski and Iwasa, 1998) and has been widely documented in many animal species including birds (Galef and White, 1998; Brown and Fawcett, 2005), fish (Coolen et al., 2003; Hill and Ryan, 2006; Alonzo, 2008), mammals (Freeberg, 2000; Galef et al., 2008), and insects (Coolen et al., 2003; Battesti et al., 2012). Mate-choice copying could serve as an efficient way to exploit social information as a quick, low-cost indication of mate quality (Pomiankowski, 1987). The idea that mate choice copying could also be present in humans (Dugatkin, 2000) has been supported by many studies (Place et al., 2010). In particular, it has been demonstrated that both men and women expressed more interest in engaging in a relationship with a potential mate if that mate was paired with an attractive partner (Yorzinski and Platt, 2010).

### 2.3. Mating as a signal of attractiveness

In the presence of mate-choice copying, the copulating behavior itself can emerge as a signal of attractiveness. It may then become advantageous to give this signal by any means available, which is not necessarily copulating with a conspecific of the opposite sex. This can lead to the appearance of DSTBs (though not DSPs). The latter idea has been proposed by Schlupp et al. (1994) to explain why it may be advantageous for male fish *Poecilia latipinna* to copulate with unisexual *Poecilia formosa*, and by Bierbach et al. (2013) to explain male homosexual behavior in *Poecilia mexicana*, in which mate-choice copying has been documented before (Andersson, 1994). In particular (Bierbach et al., 2013) have demonstrated experimentally that in *P. mexicana* males' attractiveness increases after females see them interacting sexually with either a female or another male. Note, however, that DSPs cannot appear in this way; moreover, the behavior that is being selected on is very different from the lack of interest that is central to the hypothesis proposed here.

### 2.4. Choose the choosiest: the lack of interest as indicator of attractiveness

The following method of attractiveness estimation can be used when there is sexual selection of each sex by the opposite one: not only females choose males, but also males choose females. In this case, the more attractive (say) a male is, the choosier he can allow himself to be, that is, the more attractive are the females he will choose to mate with. This means that if a female observes that a male does not choose her, she can conclude that he is able to choose other females that are more attractive, which means that he is very attractive himself: he is “hard to get.” Thus, the lack of interest emerges as a signal of attractiveness. Expressing (possibly simulating) a lack of interest is a phenomenon also known as “playing hard to get,” which has been extensively studied in humans in the psychological literature (Wright and Contrada, 1986; Eastwick et al., 2007; Jonason and Li, 2013), though it remains to be studied in other species.

### 2.5. Which method to use

A quick review of the methods for estimating attractiveness of potential mating partners outlined above shows that each of them is not only imperfect, but is nowhere close to giving a complete picture of attractiveness of each potential mate. Indeed, attractive features are rather arbitrary agreements about what is considered attractive; they do not provide any means of estimating empirically the reproductive success of the individual in the given population. Mate-choice copying is essentially an empirical estimate that is based on a very small sample (starting from samples of size 1). This is prone to large errors and high variance. Gauging the level of interest as indicator of attractiveness may indirectly take larger samples into account, but suffers from the additional bias due to imitation (“playing hard to get”).

Thus, since each method for estimating attractiveness of potential mating partners is imperfect, and since, moreover, they are to a large extent complementary, their use is limited mostly by their availability. Let us consider briefly the availability of each method. Attractive features are perhaps most highly available: all that is necessary is the ability to perceptually compare the potential mates, if only for a short time. Thus, it is enough that the population congregates around the mating time, and lives dispersedly otherwise (Shuster and Wade, 2003). Moreover, in such populations features of attractiveness may be the only way to assess the attractiveness of potential mates. This may explain why they exhibit highest levels of sexual dimorphism, including such features as bright sex-specific plumage or fins. Mate-choice copying clearly requires that the population in question lives socially in mixed-sex groups, and this appears to be the only requirement. Finally, as noted above, the level of interest as indicator of attractiveness requires mutual mate choice: both males choose females and females choose males. Mutual mate choice should arguably arise in any socially living species where each sex contributes to raring the young (Parker, 1983; Johnstone et al., 1996). In reality it is not that ubiquitous (Kokko and Johnstone, 2002), though still present in many species (see, e.g., Langmore et al., 1996; Kraak and Bakker, 1998) including our own (Gangestad and Scheyd, 2005; Stone et al., 2007). The use of the latter method of estimating attractiveness, along with the choosiness it entails and with the Fisher runaway process, is the basis for the proposed explanation of the evolutionary origins of DSPs, which we present in the next section.

## 3. The proposed explanation of the evolution of DSPs

DSPs appear as a result of the Fisher runaway process started from the lack of interest as a feature of attractiveness (choosing the choosiest). The latter emerges naturally in a population where the sexual choice is mutual: the more attractive individuals can allow themselves to be choosier, and the opposite sex uses this to estimate attractiveness. Once the “agreement” has been reached that the lack of interest signifies attractiveness, it serves as the starting point for the positive feedback loop of the Fisher runaway process: it is beneficial to select the choosiest simply because they are considered attractive. At a certain point of the runaway process the lack of interest becomes costly, as some reproductive opportunities are lost because of it: it is no longer choosiness, but a genuine lack of interest, or a sexual preference toward something different than reproductive targets. However, the runaway process continues, until this cost reaches a balance with the benefit conferred by the popularity of the trait of the lack of interest.

Note an important difference of this runaway from the classical examples. Rather than starting from a species-specific or simply random sign of popularity (such as a bright feather or a song), here it is bootstrapped by a generic, species-independent trait, that is present in any population exhibiting the right prerequisites. This explains the appearance of DSPs in different, distant species.

By the process described so far, a complete aversion to reproductive opportunities cannot develop. The aversion develops (driven by the runaway process) only inasmuch as it is counterbalanced by external forces. This can be envisaged as a balance, where on one side is the lack of interest to reproductive opportunities, while on the other side is the attractiveness of this trait. The former decreases and the latter increases the chances to reproduce. Thus, individuals exhibiting DSPs do not decrease their fitness: under stable conditions, their reproductive output does not differ from the average. Taking an example of a DSP, in this way homosexuality can appear to the extent when there is a preference toward the same sex (that guarantees a sufficient lack of interest to the opposite sex), but still some interest toward the opposite sex as well, which guarantees that the individual will reproduce given sufficient interest (or, rather, pressure) from the other side.

However, other forces can be put on the runaway “lack of interest vs. attractiveness” scale. One familiar example comes from our species. Throughout the recorded history of humanity, certain sexual preferences, such as male homosexuality, have been persecuted in many if not most societies, and remain persecuted in some to this day (Altman et al., 2012). This creates the pressure to mimic the accepted sexual behavior, such as establishing a heterosexual couple. In humans this can result in societies with universal marriage (Weinrich, 1987). In any case, whatever augments the pressure to reproduce (in the latter example, the society that discourages DSPs) acts on the “attractiveness” side of the balance, thus allowing the Fisher runaway to proceed, on the other side, and to increase further the lack of interest to reproductive opportunities, resulting in a further radicalization of the DSP.

This example should not be interpreted universally, that is, we do not claim that the same mechanism applies to all species in which DSPs are manifest, nor is it necessary for the proposed explanation of the evolution of DSPs to work. Rather, it is an illustration of how external (to sexual selection) forces can influence, and in this case, reinforce the Fisher runaway process, leading to a further development of the DSPs. At the same time, it is interesting to note that the subject of the interaction of the population as a whole with those individuals that exhibit DSPs in non-human animals has (to the best of our knowledge) not been studied, although these interactions can potentially be non-trivial and interesting in gregariously living species.

The theoretical considerations presented imply that DSPs should appear in any population living in large mixed-sex groups in which sexual selection is present and is sufficiently strong in each sex. Note that for the latter condition to hold it is necessary that each parent contributes significantly to the survival of the young. In the next section we review supporting evidence for hypothesis advanced, and discuss further research necessary to establish its validity in any given species.

## 4. Supporting evidence and directions for further research

### 4.1. DSPs and reproduction

As explained above, the suggested hypothesis for the evolution of DSPs implies that the individuals possessing this trait do not suffer a reproductive disadvantage. Moreover, in order for the individuals with DSPs to reproduce, sufficient motivation (or even pressure) should be applied to them, for example, by the members of the opposite sex. Behavioral studies can reveal whether such pressure is indeed applied, that is, whether individuals with DSPs are actively solicited more than average by the opposite sex. This kind of pressure has been documented in gees (Lorenz, 1988). For other species in which DSPs are known further studies are necessary to reveal whether this phenomenon is indeed present.

More generally, there is no strong consensus in the literature on whether DSPs affect (negatively) the reproductive outcome (Kirkpatrick, 2000). In humans, it is estimated that between 3 and 10% of men and between 1 and 4% of women are homosexual (Gelder et al., 1989). Concerning the reproductive output, A Kinsey Institute study supplies one source of data about the propensity of homosexuals to engage in heterosexual sex which could result in offspring: “Of 262 self-identified lesbian women, 75% had had sex with men since age 18 and 43% of those who had always identified themselves as lesbian had done so” (Sanders et al., 1990). It is not uncommon for gay men and lesbian women (often for religious or social reasons) to marry into a heterosexual relationship and have children (Jones, 1978). In a sample of British women (*n* = 3180), bisexuals have significantly higher fecundity to age 25 and no significant difference in lifetime fecundity when compared with heterosexuals (Baker and Bellis, 1994; Kirkpatrick, 2000). In another study, of approximately 265 homosexual and bisexual men over 30 years old in Japan, 83% have offspring (Isomura and Mizogami, 1992). Roughgarden (2004) refers to a 1994 survey which reported that 67 percent of lesbian women were mothers, compared with 72 percent of straight women (only a 5% difference). She concludes: “All in all, the data do not support uncritical acceptance of homosexuality as deleterious.”

Thus, further research is necessary to evaluate the reproductive output in individuals exhibiting DSPs as compared to the average over the population. Note that behavioral observations alone would not be sufficient, since the copulation frequency is not a sufficiently reliable indicator of the reproductive success. Furthermore, it should be noted that in human societies in which the attitude toward DSPs and behaviors has been changing in the last few generations (e.g., in the industrialized western societies), such measurements would not be conclusive, since, as explained above, negative pressure from the environment (positively) affects the reproductive outcome.

### 4.2. Derivative strategies: mimicking different preferences

If individuals with DSPs are attractive for the opposite sex, it becomes advantageous for individuals that do not have any DSP to mimic the behavior and appearance of the former in order to exploit this advantage. In other words, a parasite “*mock-DSP*” mating strategy can emerge: it exploits the reproductive benefits of a DSP, without conferring its reproductive costs. Note that imitating the lack of interest itself is just one way of mimicry, but not the only one. To see this, first note that DSPs themselves are not a way to mimic choosiness or popularity. Even though the Fisher runaway starts from the trait of lack of interest as a sign of choosiness, from the point when the trait itself turns attractive it may become completely decoupled from choosiness. In other words, members of the opposite sex may consider a DSP a feature attractive by itself, and not because they “think” it signifies choosiness. Likewise, a phenotypic or behavioral trait may become a sign of a DSP and thus a sign of popularity. It can subsequently be picked up by the Fisher runaway process and developed accordingly. As a consequence a “mock-DSP” strategy may consist in possessing this secondary trait but not a DSP. Of course just as a DSP cannot spread over the whole population, mimicking this strategy cannot replace the original strategy, but can only coexist in a balance with it. As far as we are aware, evidence of such “mock-DSP” strategies in humans exists, but only in anecdotal form.

In general, since the appearance of this strategy requires the individuals displaying DSPs to be attractive for (a significant portion of the individuals of) the opposite sex, finding this strategy in a species can be considered a proof of the hypothesis that a DSP has indeed appeared in this population by the way we describe. On the other hand, the absence of such “mock-DSP” strategies does not disprove our hypothesis, since it is not clear how large a proportion of the individuals displaying different preferences should be in order to support (or give rise to) a “parasite” strategy.

### 4.3. Dependence on the social conditions

Dependence of mating preferences on social conditions is a widespread phenomenon in animals (Rodríguez et al., 2013). However, to the best of our knowledge, the particular type of dependence that we are looking for so far has been only observed and studied in humans. Namely, we are interested in the effect of discriminative pressure exercised by the population on individuals with DSPs.

Overwhelming evidence exists that human societies have been exercising negative pressure on bearers of various different sexual preferences, most notably homosexuality, to a varying extent over different cultures. As explained above, the more the pressure the more radical should be the corresponding DSP, that is to say, the more should be the lack of interest toward members of the opposite sex capable of reproduction. This dependence can in principle be measured to verify or refute our hypothesis. However, this can be technically rather difficult: while preferences can be measured using questionnaires (such as the Kinsey scale), the answers individuals are willing to give may be affected by the attitude of the society toward the studied sexual preference, making the comparison between different populations difficult or inconclusive.

## 5. Conclusions

The hypothesis described in this paper presents a theoretical solution to an evolutionary paradox that withstood decades of both theoretical and empirical research, namely, the evolution of differences in sexual preferences. We speculate that the mechanism described is bound to produce differences in sexual preferences in any mixed-sex large population where sexual selection is present and is sufficiently strong in each sex. This opens the possibility of both theoretical and field research.

Theoretically, convincing models are needed to quantify what “sufficiently strong” means. It should be noted that the mathematical models currently employed for studying related hypotheses appear to be inadequate for modeling the phenomenon under study, and thus more sophisticated models are in order. The main complication is that the trait on which Fisher runaway is supposed to act (the interest in the opposite sex or in specific partners) is itself of direct evolutionary importance.

In the field, several ways to verify the proposed hypothesis can be envisaged. The following questions can be subjected to empirical studies aimed at establishing or refuting the validity of the proposed explanation in any given population.

- Is the sexual selection mutual (females select males and males select females)?
- Under stable environmental and populational conditions, is the reproductive outcome of individuals expressing different sexual preferences lower than the population average?
- Are individuals displaying DSPs solicited more actively by the opposite sex?
- Is the “mock-DSP” mating strategy present in the given sex?
- In case several populations are available, in which, under otherwise similar conditions, some populations exercise negative pressure against individuals with DSPs: In the populations that do exercise the negative pressure, are the DSPs more radical (further aversion toward the opposite sex)?

The first two questions are pre-requisites: unless the first one is answered positively and the second negatively, the proposed explanation is not valid for the population in question. A positive answer to any of the rest of the questions provides a strong support for our explanation, and a negative is an evidence against it.

### Conflict of interest statement

The authors declare that the research was conducted in the absence of any commercial or financial relationships that could be construed as a potential conflict of interest.

## Footnotes

^1^The less we love a woman \\ the easier 'tis to be liked by her, \\ and thus more surely we undo her \\ amid seductive toils. *Translated by Vladimir Nabokov, 1964*.
